# Supplementary material for: Promises and pitfalls of using LLMs to identify actor stances in political discourse
Source: PLoS One. 2025 Nov 19;20(11):e0335547. doi: 10.1371/journal.pone.0335547 (PMC12629487; doi:10.1371/journal.pone.0335547)
Supplement: Attachment — Submitted filename: response_letter.pdf [file pone.0335547.s001.pdf]

Responses to reviewer comments

Manuscript: Promises and pitfalls of using LLMs to identify actor stances in political discourse

Revision 1

October 2, 2025

Editors' comment

I think the three reviewer's have provided very useful comments and suggestions for the submission. I think the authors should read each of the comments carefully and respond to each in a revised version.

Reviewer 1

This manuscript examines the application of Large Language Models to stance detection in political discourse. The research is important and timely, as it systematically evaluates different LLMs for a specific NLP task with real-world applications. As more researchers consider incorporating LLMs into their analytical workflows, empirical assessments of their capabilities on domain-specific tasks provide valuable guidance. The authors test LLMs on German newspaper content, which offers a more realistic assessment than using standard benchmark datasets. However, this approach introduces its own limitations regarding generalizability beyond German media discourse on transportation policy. The following sections reports my considerations and improvement areas.

Methodological Considerations

The evaluation approach tests various prompt chain structures, compares models of different sizes, and examines performance across statements. This multifaceted approach is methodically sound, though the number of tested variables creates challenges for isolating key factors driving performance differences.

Reviewer comment 1:
The paper includes entity masking as a variable and documents inter-annotator agreement scores, which provides useful context. However, the modest agreement score (averaging 0.55) raises questions about the reliability of the ground truth data against which LLM performance is measured.
Answer:
The reviewer raises one of the key methodological issues in our analysis, namely the initially low average IAA of 0.55 (and 0.62 for batches annotated by two annotators) for our evaluation set and what this means for our analysis. It is certainly the case that our analysis stands on shaky grounds if we cannot take our evaluation set as something resembling a ground truth (however fraught this term is in social science context) to evaluate against. We have updated the paper to better reflect this situation in three main ways: First, we highlight more prominently that the final evaluation set is always the product of a procedure where for all annotations with annotator disagreement, a consensus solution was reached in a discussion with the whole annotator team. We thus feel relatively confident about the final annotation quality of the evaluation set used. Second, we further emphasise that the fact that the annotation task is indeed a challenging task for humans and thus the performance of the classification model should be really seen in this context. Third, we have now reached an agreement with the original providers of the data and are able to fully share the original 1710 paragraph texts, which should also further enable replication and scrutiny.

Reviewer comment 2:
A methodological gap is the lack of detailed information about dataset retrieval. While the authors mention sourcing paragraphs from SwissdoxLiRI, a Swiss media corpus, they provide insufficient detail about search criteria, selection parameters, or sampling strategy. Could the authors add this information, at least, in the Appendix?
Answer:
The following description of the dataset retrieval was added to the manuscript. As mentioned in response to the reviewer's earlier comment, we are now also able to share the complete evaluation data including the full paragraph texts annotated.

> ***New text:*** *"The articles, from which paragraphs were drawn, are sourced from online and print articles available through Swissdox@LiRI, a Swiss media corpus accessible to the authors. Articles originate from three major Swiss news publications (Tages-Anzeiger, Neue Zürcher Zeitung, 20 Minuten). In their political orientation, our media sources range from the more right-leaning Neue Zürcher Zeitung (NZZ) to the left-leaning Tages-Anzeiger (Udris et al. 2023). The evaluation dataset covers paragraphs sampled randomly from a subset of paragraphs from all articles published in the newspapers containing 1)Zürich-related 2) sustainable transport topics, 3) with at least one organizational entity mentioned, based on a multi-stage analysis pipeline documented in BLINDED. Paragraphs span a time span of 13 years, from the beginning of 2010 until the end of year 2023. "*

Other Critical Points

Reviewer comment 3:
The paper identifies performance variations across different statements but offers limited analysis of these differences. The consistent finding that stances toward air travel reduction were better detected merits deeper exploration of potential causal factors. I think that the paper lack of qualitative error analysis. It would benefit from a more systematic examination of where and why LLMs fail at stance detection. Such analysis could reveal patterns in misclassifications, identify challenging linguistic features, and provide practical guidance for researchers applying similar methods to other contexts. Also a brief analysis of differences between LLM and human stance detection remain underexplored.
Answer:
We agree with the reviewer that a qualitative analysis on misclassifications deepens our paper's findings. We thus did a thorough error analysis on classification error by the LLMs per statement, LLM, prompt chain and entity masking. The results chapter in the revised paper was hence extended with the section Error analysis in which we elaborate on four different cases of misclassifications.

Reviewer comment 4:
I found also confusing the absence of a dedicated limitations section. While limitations are mentioned throughout the manuscript, this lack makes it difficult for readers to fully appreciate the constraints of the study, considering the methodological nature of the study. A consolidated discussion of methodological, conceptual, and practical limitations would enhance the paper's scholarly rigor and utility for other researchers.
Answer:
We agree with the reviewer that a dedicated limitation section would improve the paper and have added such a section.

Broader Implications

Reviewer comment 5:
The research has implications for social science methodology, suggesting that researchers with limited computational resources might leverage smaller models with well-designed prompting strategies. However, the performance levels achieved (F1 scores up to 0.73 in the best case) indicate substantial room for improvement before such methods could reliably replace human coding. The authors acknowledge the reductive nature of mapping complex political positions to discrete categories. This tension, inherent in computational approaches to discourse analysis, deserves more critical examination regarding the validity of LLM-based stance detection for drawing substantive political science conclusions.
Answer:
By reworking the manuscript, we found space to add discussion in different places (starting from the abstract, in a dedicated limitations section, as well as in the conclusion), to further reflect on this point. We now do more strongly emphasize that our results indicate that these tools are probabably not at a point where they would win out often in a careful weighing of their application versus established valid approaches, such as manual annotation. We emphasize that key for applied political science research is to honestly weigh options every time, including the fact that domain-specific test sets will need to be annotated in any case. We also point more directly now to the very valid option of simply annotating a representative sample as an alternative that should always be considered.

Reviewer comment 6:
Furthermore, the paper could benefit from a more explicit discussion of how the findings contribute to the broader field of political discourse analysis beyond the technical achievement. The authors could more thoroughly connect their methodological insights to substantive questions in political communication, ideology formation, or public opinion research.
Answer:
We considered this suggestion by the reviewer for some time. In the end, we had to weigh the trade-off between a methodologically focused article and introducing more substantive content. We eventually decided to focus the article on the methodological contribution, especially because we have made more substantive contributions applying (but not discussing and testing at length in the way this article gives us the room to do) the approach. We still discuss the relevance of the approach however, as well as focusing our recommendation in a very field-specific way, cautioning against weighing the hype around LLMs versus it actually improving the likelihood of making valid substantive contributions.

Reviewer comment 7:
Also a discussion of ethical implications is missing. The authors briefly mentioned to use opensource LLMs due to ethical concerns and acknowledge bias concerns through their entity masking experiments. In general, given the political nature of stance detection, the manuscript would be strengthened by a more robust discussion of potential biases and ethical implications. The use of LLMs to classify political positions raises important questions about algorithmic fairness, the potential amplification of existing biases in political discourse, and the ethics of automated categorization of political actors' stances. A deeper engagement with these ethical dimensions would enhance the paper's contribution.
Answer:
We agree with this comment and have added 1) some additional language referencing the need to be especially careful with applications in politically sensitive contexts, pointing toward ongoing investigations in political "leanings" of models and 2) are more assertative in emphasizing that LLM application should be honestly weighted against existing approaches, even explicitly pointing toward manual annotation of represenative samples in the abstract.

Overall Assessment

In general, I found this manuscript very fascinating and helpful for social scientists who want to introduce LLMs in their research, in particular for content annotation tasks.

Reviewer 2

The authors set out to explore zero-shot stance detection on 1,710 German newspaper paragraphs about sustainable urban transport, comparing seven prompt-chain strategies across four variants of Llama3 models. They report a top weighted-macro F1 of 0.73 and highlight the importance of prompt design, model size, and statement-specific performance differences.

Reviewer comment 8:
However, the manuscript offers little in the way of novel methodological contribution. Many of the prompt-chain ideas (e.g., summary-then-classify, hierarchical checks) closely mirror existing "chain-of-thought" or multi-stage prompting techniques already explored in recent LLM literature [16,17,26], yet the authors neither clearly delineate how their chains differ nor benchmark against those prior works. Without a compelling new algorithmic insight or a head-to-head comparison with fine-tuned or few-shot baselines, the paper reads as an incremental application rather than a substantive advance.
Answer:
We agree with the reviewer in this respect and are now even more transparent that this study is an incremental application, which we now state as such at the end of the state of the art section. Additionally, we provide further reasoning on why this is not a bad thing to contribute as a data point in the ongoing discourse around using LLMs in applied social science research in the discussion and (new) limitations section.

Reviewer comment 9:
The annotation methodology also raises serious questions. Reported inter-annotator agreements ranged from 0.41 to 0.63 (mean 0.55), underscoring the inherent ambiguity of the task even for humans. Yet the authors do not analyze how this noise propagates into model evaluation, nor do they provide a human-performance benchmark for comparison. Critically, they exclude their initial batch used to build the codebook, which may bias the remaining "gold" data toward easier cases.
Answer:
The reviewer raises one of the key methodological issues in our analyis, namely the initially low average IAA of 0.55 for our evaluation set and what this means for our analysis. It is certainly the case that our analysis stands on shaky grounds if we cannot take our evaluation set as something resembling a ground truth (however fraught this term is in social science context) to evaluate against. We have updated the paper to better reflect this situation in three main ways: First, we highlight more prominently that the final evaluation set is always the product of a procedure where for all annotations with annotator disagreement, a consensus solution was reached in a discussion with the whole annotator team. We thus feel relatively confident about the final annotation quality of the evaluation set used. Further, we now make it more clear that the initial "batch0" used to get annotators acquainted with the domain and to build the codebook did not differ in any substantive way from the next batches that were annotated. Second, we further emphasise that the fact that the annotation task is indeed a challenging task for humans and thus the performance of the classification model should be really seen in this context. Third, we have now reached an agreement with the original providers of the data and are able to fully share the original 1710 paragraph text, which should also further enable replication and scrutiny.

Reviewer comment 10:
Furthermore, the annotation codebook—while included—lacks sufficient detail on borderline examples, making replication and assessment of labeling quality impossible.
Answer:
We have supplied the codebook as used during the annotation process and cannot retroactively add detail to it, as this would not be truthful to how the annotation was conducted. The possibility for replicating the labeling based on the codebook is an empirical question in our view.

Reviewer comment 11:
Evaluation is similarly limited. The study is confined to a single domain (sustainable transport) and one language (German), with no experiments to test generalizability across topics, styles, or languages. There is no statistical testing of differences between prompt chains or models, and no thorough error analysis to reveal systematic failures (e.g., on sarcasm or implicit statements).
Answer:
The reviewer correctly points out the considerations a reader of our manuscript needs to make in inferring potential generalizability. We now stress more prominently in a limitations section of the discussion that this is an example of a domain-specific, scoped analysis, which we see as as valuable at the current time as reports on more general approaches and generic benchmarks, because this is how social scientists actually working in domain-specific context will use these tools. We do report statistical tests on the influence of entity masking and type of statements. Also based on comments by another reviewer, we now include a more thorough error analysis (see section Error analysis in the Results chapter).

Reviewer comment 12:
The absence of any computational-cost or latency measurements further hinders assessment of real-world feasibility.
Answer:
The reviewer raises an important point regarding applications concerned with speed or latency, which we did not consider, as our typical use case envisioned for this type of analysis and tooling was not overly concerned with latency or computational cost. We now discuss this in the limitation section added due to comments by another reviewer.

Reviewer comment 13:
Finally, the manuscript's presentation suffers from overlong, jargon-laden passages and insufficient clarity on critical details: exact prompt templates are buried in the appendix;
Answer:
We agree with the reviewer that an effort should be made to avoid unnecessary jargon whenever possible and have given the manuscript another read with attention to remove it. However, as the reviewer did not indicate specific passages, we might have missed out on some instances. The choice to include only one prompt chain visualization in the main text, rather than all seven, was made to avoid overloading the main text with large visualizations, each of which takes up half a page. To ensure understanding about the prompt chains, we have described all prompt chains in prose within the main text (p. 6). The visualizations of the remaining six prompt chains are provided in the Appendix for readers who prefer a more comprehensive and visual understanding.

Reviewer comment 14:
hyperparameter settings, inference details, and code-release links are not readily accessible;
Answer:
This should be rectified in the latest version of the accompanying zenodo repository, which enables full replication of the evaluation setup, including all evaluation data, as well as now containing the code needed for running the exact inference script used to produce every evaluation data instance. Generally, for inference, default parameter settings were used (documented in the /inference folder of the replication repository), as our evaluation matrix was anyhow stretching the limits of feasibility already.

Reviewer comment 15:
and key tables (e.g., per-class metrics) are omitted.
Answer:
We have now made error analysis metrics available as a sortable, interactive table, which we agree provides additional fine-grained detail.

Reviewer comment 16:
Also, the figures are missing.
Answer:
We can empathize with the reviewer, given that reading a manuscript without figures is not a particularly nice experience. However, our hands were tied in this, as according to PLOS ONE submission guidelines, the figures must be excluded in the uploaded manuscript and handed in separately. While the figures made it to other reviewers of the paper, they seem to not have made it to this specific reviewer. We hope this has been rectified in this resubmission.

Reviewer 3

This paper explores zero-shot stance detection using German LLaMA3-based LLMs applied to political discourse in German newspaper texts. It proposes several prompt chain strategies and evaluates them across five policy-related statements, offering insights into model performance variation and the utility of summarization and entity masking.

Strengths: 1. Timely and relevant topic bridging NLP and political discourse analysis. 2. Use of real-world, manually annotated German-language data. 3. Systematic evaluation of multiple prompt chain strategies. 4. Open-source code and data enhance reproducibility.

Weaknesses:

Reviewer comment 17:
1. Limited Language Scope: The study only uses German data and models; cross-lingual validation or multilingual experiments would improve generalizability.
Answer:
The reviewer correctly points out the considerations a reader of our manuscript needs to make in inferring potential generalizability. Throughout the manuscript, but especially in the newly added limitations section, we now stress more prominently that this is an example of a domain-specific, scoped analysis, which we see as as valuable at the current time as reports on more general approaches and generic benchmarks, because this is how social scientists actually working in domain-specific context will use these tools.

Reviewer comment 18:
2. Unclear Prompt Design Rationale: Prompt chains are heuristically designed without sufficient justification or ablation.
Answer:
The reviewer is right that the definition of prompt chains did only occur to a part in a deductive manner, mostly inspired by previous multi-stage prompt approaches to stance detection. We added text indicating this in the section introducing the prompt chains

> ***New text:*** *" The specific prompt chains were formulated to explore four main concepts, in different combinations: 1) the inclusion of a summary step, 2) alternating orders of checks for relevance, 3) the inclusion of a dynamic, statement specific prompting step and 4) using constrained grammar, basing classification on generating text that is not a direct label. "*

Reviewer comment 19:
3. Missing Bias Analysis: No examination of potential LLM bias, despite using politically sensitive content.
Answer:
We more strongly emphasize the specific avenues for bias we can investigate in the paper (especially regarding entity masking) and added some paragraphs on general avenues for bias in politically sensitive investigations.

Reviewer comment 20:
4. Low Annotation Agreement: Inter-annotator agreement is modest (mean IAA = 0.55), raising questions about label clarity and task definition.
Answer:
The reviewer raises one of the key methodological issues in our analyis, namely the initially low average IAA of 0.55 (and 0.62 for batches annotated by two annotators) for the initial basis of our evaluation set (although the final evaluation dataset underwent another round of reviewing) and what this means for our analysis. It is certainly the case that our analysis stands on shaky grounds if we cannot take our evaluation set as something resembling a ground truth (however fraught this term is in social science context) to evaluate against. We have updated the paper to better reflect this situation in three main ways: First, we highlight more prominently that the final evaluation set is always the product of a procedure where for all annotations with annotator disagreement, a consensus solution was reached in a discussion with the whole annotator team. We thus feel relatively confident about the final annotation quality of the evaluation set used. Second, we further emphasise that the fact that the annotation task is indeed a challenging task for humans and thus the performance of the classification model should be really seen in this context. Third, we have now reached an agreement with the original providers of the data and are able to fully share the original 1710 paragraph texts, which should also further enable replication and scrutiny.

Reviewer comment 21:
5. Overstated Conclusions: Claims about general applicability are not fully supported given the narrow empirical setup.
Answer:
We hope to have addressed this concern by focusing much more strongly on the type of generalizability our study cannot offer, specifically in the limitation section.

Reviewer comment 22:
6. Figures Lack Clarity and Professionalism: Several figures (e.g., Fig. 11–13) are cluttered, use inconsistent formatting, and do not follow standard academic design conventions, making them hard to interpret.
Answer:
We have added additional white space to the figures to unclutter them.

Overall, the paper addresses an important problem and has potential. However, a major revision is needed to improve comparative evaluation, clarify methodology, and broaden the empirical scope.
